# Phospholipids and sports performance

**DOI:** 10.1186/1550-2783-4-5

**Published:** 2007-07-25

**Authors:** Ralf Jäger, Martin Purpura, Michael Kingsley

**Affiliations:** 1 Increnovo LLC, 2138 E Lafayette Pl, Milwaukee, WI 53202, USA; 2 Department of Sports Science, University of Wales Swansea, Singleton Park, Swansea, SA2 8PP, UK

## Abstract

Phospholipids are essential components of all biological membranes. Phosphatidylcholine (PC) and Phosphatidylserine (PS) are Phosphatidyl-phospholipids that are required for normal cellular structure and function. The participation in physical activity often challenges a variety of physiological systems; consequently, the ability to maintain normal cellular function during activity can determine sporting performance. The participation in prolonged intense exercise has been shown to reduce circulatory choline concentrations in some individuals. As choline is a pre-cursor to the neurotransmitter Acetylcholine, this finding has encouraged researchers to investigate the hypothesis that supplementation with PC (or choline salts) could enhance sporting performance. Although the available data that evaluates the effects of PC supplementation on performance are equivocal, acute oral supplementation with PC (~0.2 g PC per kg body mass) has been demonstrated to improve performance in a variety of sporting activities where exercise has depleted circulatory choline concentrations. Short term oral supplementation with soy-derived PS (S-PS) has been reported to attenuate circulating cortisol concentrations, improve perceived well-being, and reduce perceived muscle soreness after exercise. More recently, short term oral supplementation (750 mg per day of S-PS for 10 days) has been demonstrated to improve exercise capacity during high intensity cycling and tended to increase performance during intermittent running. Although more research is warranted to determine minimum dietary Phospholipid requirements for optimal sporting performance, these findings suggest that some participants might benefit from dietary interventions that increase the intakes of PC and PS.

## Background

The functional ingredients of Lecithin are Phosphorus containing lipids called Phospholipids (PL). Hundreds of different PL molecules have been described, but the major types are the phosphatidyl-phospholipids. They consist of two fatty acids groups that are connected through a glycerol backbone to a Phosphate ester group. Phospholipids are essential building blocks of all biological membrane systems and act not only as structural molecules but also as dynamic, functionally important components of cells [1].

Phosphatidylcholine (PC) is a major constituent of all cellular membranes and its presence is necessary for normal function. Phosphatidylcholine builds a continuous membrane matrix, providing precise fluidity, charge distribution and electronic character that enzymes and other membrane molecules need to carry out their functions. Supplies of PC are needed to support membrane expansion as the cells grow, for membrane renewal, and for the regeneration of the cells' membrane system following damage. Phosphatidylcholine also operates in the liver, lungs, gastro intestinal tract and kidneys as a surface-active wetting-agent to coat cell linings. Phosphatidylcholine supplementation have been found to support healthy cholesterol levels [2], liver [3] and brain functions [4] and due to the essential nutrient choline, which comprises about 15% of the PC molecule, PC supplementation might be beneficial for endurance athletes [5].

Phosphatidylserine (PS) [6] is present in all biological membranes of animals, higher plants and micro organisms. In humans, PS is most concentrated in the brain where it comprises 15% of the total phospholipid pool. In other human tissue the ratio of PS in the Phospholipid pool varies: lungs (7.4%), testes (6.4%), kidneys (5.7%), liver (3.8%), skeletal muscle (3.3%), heart (3.2%) and blood plasma (0.2%). The total body PS pool is estimated to be approximately 60 grams, with 30 grams in the brain and 30 grams in the rest of the body. The average daily PS intake from the diet in western countries is estimated to be 130 mg [7]. Phosphatidylserine is located mainly in the internal layer of the cell membrane and has a variety of unique structural and regulatory functions. Phosphatidylserine is involved in governing membrane fluidity and therefore in the regulation of biological cell activities. As PS exhibits direct and indirect interactions with integral and membrane-associated proteins, it modulates the activity of receptors, enzymes, ion channels and signalling molecules. Phosphatidylserine supplementation improves brain functions that tend to decline with age [8]. Recent studies indicate that PS supplementation might be beneficial for children with attention deficit hyperactivity disorder [9] and young people suffering from mental [10] and/or physical stress [11].

### Phosphatidylcholine

#### Intense exercise lowers choline levels

Muscle contractions are induced by signals carried along cholinergic nerves to the muscle fiber. Acetylcholine, the signaling molecule or neurotransmitter, is synthesized from choline. The concentration of free choline can influence the rates in which acetylcholine is synthesized and released [12-15]. Consequently, a reduction in free choline during intense physical activity might reduce acetylcholine release and thereby affect endurance and performance.

**Figure 1:**
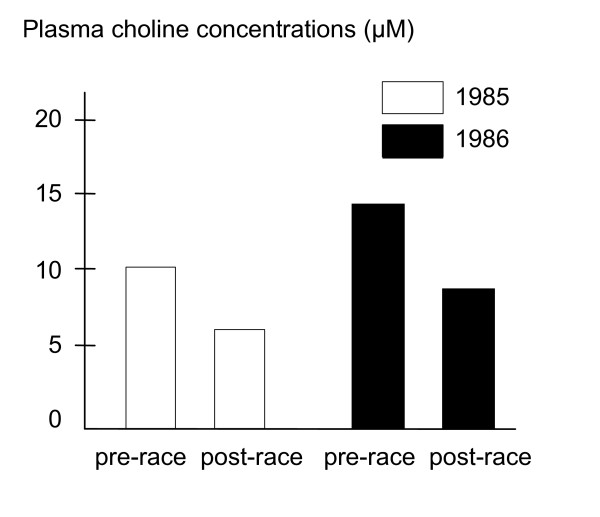
Runners in the Boston marathon showed a decrease in plasma choline levels of about 40% [16,17].

Intense exercise of long duration relies extensively on the muscle contraction signaling pathway. Strenuous exercise has been reported to result in decreased choline concentrations within the blood. For example, runners of the 1985 and 1986 Boston Marathon showed a 40% drop in plasma choline levels [16,17] (see figure 1); reductions in circulatory choline have also been reported in cyclists [5,18], and runners [18-20].

### Phosphatidylcholine Supplementation Increases Choline Levels

**Figure 2:**
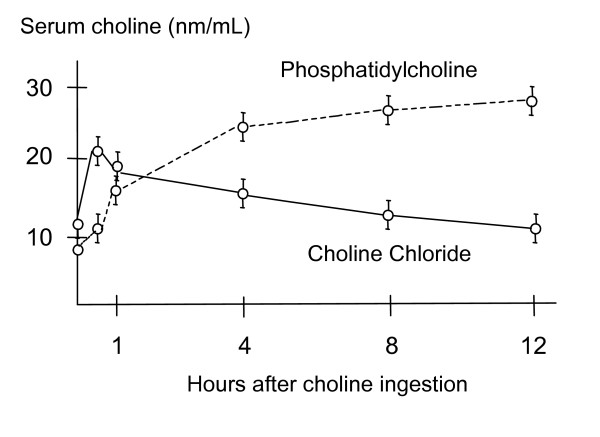
Serum choline concentrations after ingestion of 2.3 g choline as choline chloride or PC [21,22].

Several studies have shown that choline supplementation is able to significantly increase plasma choline levels [21,22]. Phosphatidylcholine is 12 times more effective than inorganic choline salts at raising human blood choline levels after 24 hours [21,22]. Following the ingestion of choline chloride, serum levels reach their peak (86% above normal levels) after approximately 30 minutes and return to normal values within 4 hours ^21^. Ingesting PC resulted in higher peak serum choline concentrations (265% above normal levels) and these levels remained elevated for 12 hours [21] (see figure 2).

The oral ingestion of inorganic choline salts (e.g. choline chloride, choline citrate, choline bitartrate) leads to losses of approximately 60% of the available choline, through conversion to trimethylamine by intestinal bacteria [23]. The same amount of choline ingested in the form of PC produces only one third of the trimethylamine [24]. High amounts of trimethylamine in the gastrointestinal tract can produce an offensive, fishy body odor.

### Phosphatidylcholine Supplementation Prior to Activity can Prevent Decline of Choline Levels and might improve Performance

#### Cycling

**Figure 3:**
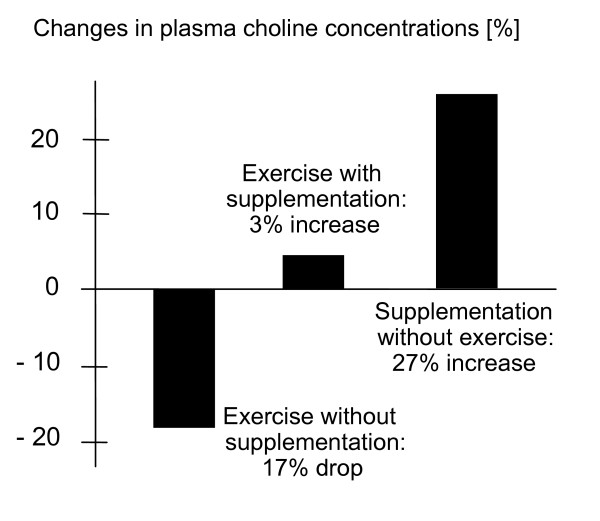
Influence of PC supplementation on plasma choline levels [5].

Von Allwörden suggested that choline supplementation prior to exercise can prevent the decrease in choline levels from hard physical stress and that this could affect performance [5]. Ten top level triathletes performed two sets of two hour cycling exercise at an average speed of 35 km per hour. The participants received either a placebo or 0.2 g PC (90%) per kg body mass. Phosphatidylcholine supplementation without exercise led to an increase in plasma choline concentrations of approximately 27%. Exercise without PC supplementation decreased the plasma choline concentrations in the triathletes by an average of 17%. When PC was given one hour before exercise, average plasma choline concentrations remained at the same level as the initial values (see figure 3).

Von Allwörden reported similar findings from a double-blind, placebo-controlled, crossover pilot trial. Well-trained endurance athletes performed a cycle ergometer test for 2 hours. Subjects received either a placebo or 0.2 g PC (90%) per kg body mass at breakfast 3 hours before cycling at 1 Watt per kg body mass [18]. Plasma choline values decreased after 2 hours of exercise by 38%. When supplemented with PC, the average plasma choline levels remained at the same level as the starting values.

**Figure 4:**
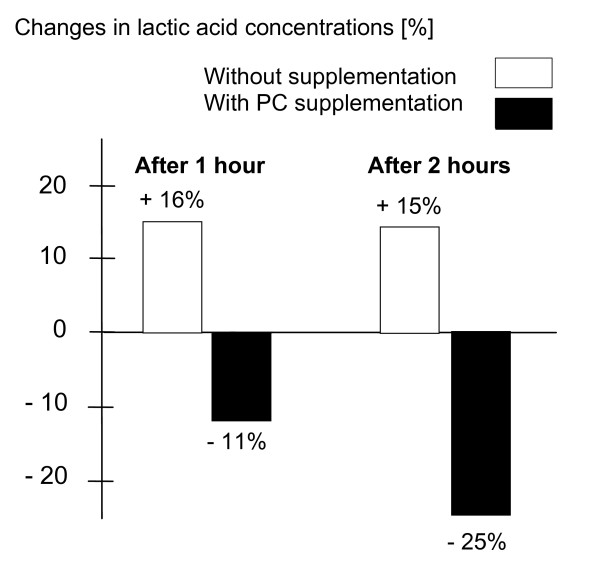
Influence of PC supplementation on lactic acid concentrations during exercise [18].

Lower steady state heart rate, without an increase in blood lactate concentration, at specific exercise intensities indicates more efficient use of aerobic metabolism [25]. The rate at which heart rate and blood lactate concentration return to baseline values following exercise reflect the effectiveness of the recovery processes. Lactic acid concentrations after 1 hour (break for taking blood samples) and after 2 hours of exercise were increased by about 15% without supplementation; however, PC supplementation led to 11% decrease after one hour and a 25% decrease after 2 hours of exercise [18] (see figure 4).

**Figure 5:**
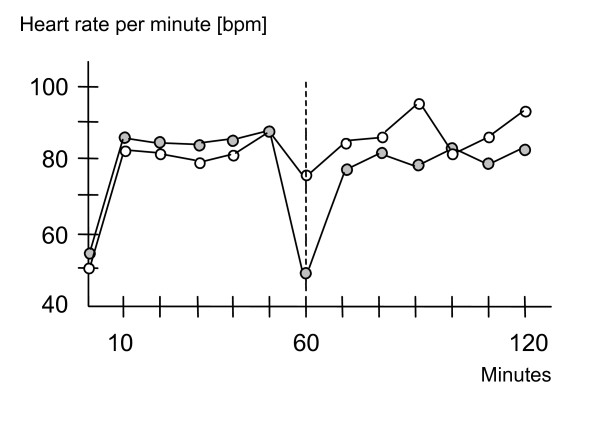
The effects of PC supplementation on mean heart rate during ergometer training [18].

Phosphatidylcholine supplementation led to a decrease in heart rate during the break for taking blood samples after one hour of exercise [18] (see figure 5). The more rapid return of heart rate to normal values after supplementation with PC when compared to placebo suggests that increasing the availability of choline improved the recovery following exercise. These findings confirm Mies et al.'s placebo-controlled study on Lecithin (a mixture of PC, PS and other PLs) in which supplementation resulted in improved recovery in a bicycle ergometer trial [26,27].

#### Long Distance Runners

Von Allwörden investigated the effects of either placebo or 0.2 g PC (90%) per kg body mass on adolescent runners (aged 14–20 years). The subjects performed cross-country races of between 30 and 60 minutes, depending on age. Phosphatidylcholine supplementation resulted in an 18% increase in plasma choline levels with and a 54% increase without exercise; however, runners without PC supplementation did not deplete plasma choline levels [5].

In a follow-up trial well-trained endurance athletes (aged 25–28 years) were running with a velocity of 12 km per hour for 2 hours. Subjects received either 0.2 g PC (90%) per kg body mass at breakfast 3 hours before exercise or did not receive a controlled diet [18]. Plasma choline values decreased after 2 hours of running by an average of 55%. When supplemented with PC average plasma choline levels increased during three hours of rest. Physical stress lowered choline levels by 41%; however, final values were still higher than initial values. Lactic acid values with PC supplementation were lower in 8 out of 10 runners and time to recover was 12% shorter.

More recently, Buchman et al. reported that supplementation with PC, in the form of lecithin, attenuated the exercise-induced reduction of plasma choline concentrations in marathon runners when compared to supplementation with a placebo [28]. Actual race times were not different from predicted race times and the authors concluded that supplementation failed to improve performance. This comparison should be viewed with caution as race times during marathon running are difficult to predict due to variations in courses and environmental conditions.

### Studies with choline salts

In a double-blind, crossover, placebo-controlled clinical trial ten long distance runners received either 2.8 g choline citrate or placebo 1 hour prior to and again after completing 10 miles of a 20 mile run [19]. Plasma choline levels were reported to have increased in the choline supplemented group, whereas they decreased in the placebo group after strenuous exercise. The mean run time was found to be significantly shorter when runners were supplemented with choline when compared to placebo, being 153.7 minutes and 158.9 minutes, respectively.

In clinical trials, in which athletes did not deplete choline during exercise, choline supplementation did not result in a delay of fatigue [29-31].

Spector at al. investigated the relationships between plasma choline and fatigue during supramaximal brief and submaximal prolonged cycling activities [29]. In a placebo-controlled trial choline bitartrate supplementation resulted in an increase in choline plasma levels. Neither group depleted choline during either brief or prolonged cycling training. Fatigue times and work performed were similar under both conditions in each group. It was concluded that choline was not depleted in either condition nor did choline supplementation delay fatigue.

Deuster et al. investigated the effects of choline supplementation on treadmill exercise in a double-blind crossover study [30]. Choline supplementation significantly increased plasma choline concentrations. Exhaustive physical activity did not deplete circulating choline levels and consequently did not affect physical or cognitive performance.

These findings confirm Warber et al.'s double-blind crossover study on choline citrate in which soldiers did not deplete plasma choline levels and did not delay fatigue during a four hour load carriage treadmill exercise [31].

### Phosphatidylserine

Overtraining is a natural hazard of competitive sports. The consequences of this condition can include decreased performance, injury, depressed immunity and psychological depression [32,33]. A serious corollary of overtraining in young female athletes is its propensity to decrease bone density [34]. During early stages of overtraining, such as when you work too hard during one or two exercise sessions, muscles become sore, resting and submaximal exercise heart rate and cortisol levels increase and testosterone levels fall. The body has difficulties in adjusting, but usually recovers with a few days' rest. Chronic overtraining often creates a disturbance in the ratio between the anabolic hormone, testosterone, and the catabolic hormone cortisol.

Phosphatidylserine was historically obtained from bovine cortex (BC-PS). Due to the potential transfer of infectious disease, this supplement is now considered unsuitable. More recently, soy-derived phosphatidylserine (S-PS) has become accepted as a safe alternative. Soy-PS is commercially available as an oral supplement, with recommended daily doses usually ranging from 100 to 500 mg.

Several double-blind, placebo-controlled clinical trials have been performed to investigate whether phosphatidylserine supplementation (BC-PS and S-PS) prior to exercise can prevent the exercise-induced increase in cortisol levels and improve other markers related to hard training.

### Cycling

**Figure 6:**
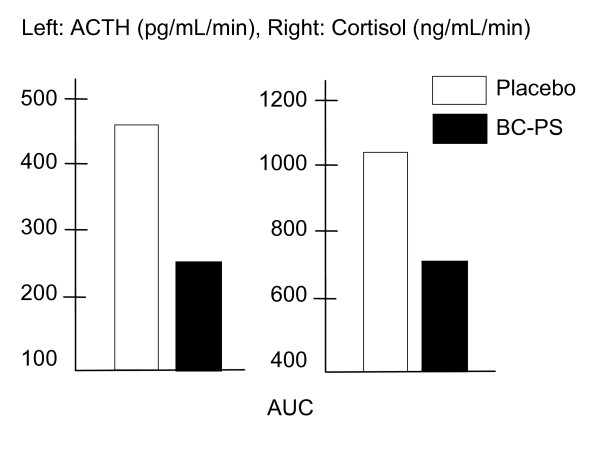
PS supplementation significantly suppresses ACTH and cortisol responses to exercise [11].

Eight non-physically trained healthy male volunteers (aged 24–42 years) underwent two exercise trials, composed of three distinct stages of cycle ergometry. The subjects received intravenously either placebo or BC-PS 10 minutes before exercise. As expected, physical exercise resulted in a significant increase in ACTH and cortisol levels after administration of a placebo. Bovine cortex-PS supplementation significantly suppressed ACTH and cortisol responses to exercise [11] (see figure 6).

In a double-blind, randomized, placebo-controlled, cross-over design, Monteleone et al. [35] showed that PS supplementation suppressed cortisol and ACTH responses to staged cycling exercise. Compared to placebo, cortisol levels were 30% lower (800 mg S-PS), demonstrating that PS supplementation can lessen the severity of stress responses to exercise.

Kingsley et al. [36] recently reported a double-blind, placebo-controlled clinical trial where fourteen active males completed staged intermittent exercise on three separate occasions, (a familiarization trial followed by two main trials that were separated by approximately 16 days). The staged intermittent protocol consisted of three 10-min stages of cycling at 45, 55 and 65% VO_2max _followed by a final bout at 85% VO_2max _that was continued until exhaustion. The exercise time to exhaustion was used as a measure of exercise capacity. After completing the first main trial the subjects consumed either 750 mg of S-PS per day or a placebo for 10 days. The main finding was that supplementation influenced exercise capacity at 85% VO_2max_. The group that received S-PS increased exercise times to exhaustion by 29 ± 8%, while the exercise times to exhaustion did not change following supplementation with placebo. Nevertheless, supplementation did not significantly influence oxygen kinetic responses (MRT_on _or MRT_off_), substrate oxidation, cortisol, and feeling states during the trial.

### Weight-Training

**Figure 7:**
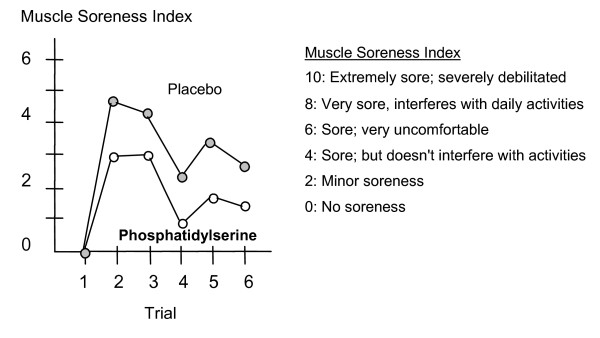
PS supplementation significantly improves muscle soreness [37].

A 1998 study conducted at California State University investigated the effect of PS on hormone levels, muscle soreness and feelings of well-being when administered to experienced weight-trained athletes [37]. During the two-week training period, 11 weight-trained athletes (aged 22.8 ± 3.4 years) consumed either a placebo or 800 mg of S-PS per day (double-blind, cross-over design). The weight training program was designed to overtrain the subjects. Subjects were instructed to use as much weight as possible for each exercise and to train as hard as possible for 4 days a week during each 2 week training period. Subjective measures, such as perception of well-being and muscle soreness were recorded throughout the training period. Athletes had less muscle soreness when they were taking S-PS compared to the placebo (see figure 7).

**Figure 8:**
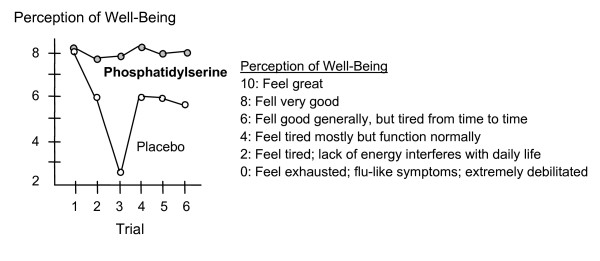
PS supplementation significantly improves well-being [37].

In addition, subjects had an improved perception of well-being when taking S-PS, this was particularly evident after the first week of training (see figure 8).

### Running Activities

**Figure 9:**
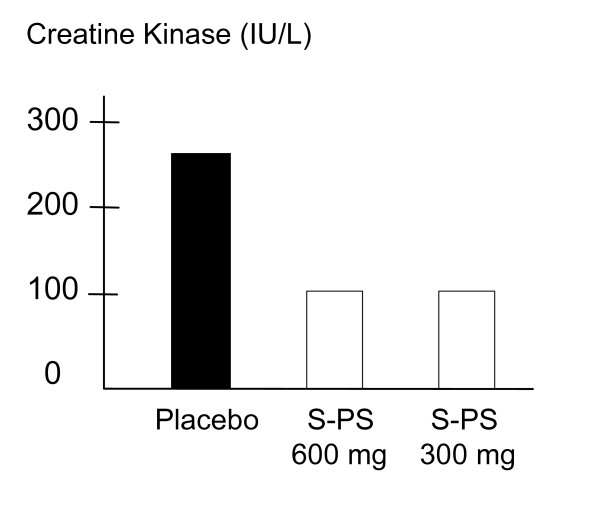
PS supplementation reduces circulatory Creatine Kinase activities [38].

A study conducted at the St. Cloud State University showed that S-PS supplementation significantly lowered creatine kinase (CK) activities in the circulation, a generally accepted indicator of cell membrane damage and necrosis of the muscle fiber, 24 hours after exercise [38]. Twelve trained runners took either 300 or 600 mg S-PS per day or a placebo for 15 days, then performed a 90-minute run on the 15^th ^day in this double-blind crossover study. In healthy rested muscle, CK is contained within the muscle plasma membrane. Intense exercise and activities, where muscle damage occurs, causes an increase in CK activities within the plasma and serum. PS supplementation was reported to reduce muscle damage in trained runners compared to placebo (see figure 9).

A more recent double-blind, randomised, placebo-controlled, crossover study investigated the effects of S-PS supplementation on markers of muscle damage, inflammation, muscle soreness and oxidative stress following eccentric exercise-induced muscle damage [39]. Independent of supplementation with S-PS (750 mg per day for 10 days) or placebo, downhill running induced similar increases in circulating CK activities, myoglobin concentrations, interleukin-6 concentrations and hydroperoxide concentrations.

Kingsley et al. [40] investigated the effects of oral supplementation with 750 mg per day of S-PS for 10 days on oxidative stress caused by exhaustive intermittent running that was designed to simulate the movement patterns of soccer with the addition of a performance run at the end of the protocol. Although supplementation did not influence lipid peroxidation (as measured by hydroperoxide concentrations), muscle damage (as measured by circulatory CK activities and myoglobin concentrations) and perceived muscle soreness, supplementation with S-PS tended to improve sprint and exercise performances when compared to placebo. This double-blind, placebo-controlled clinical trial demonstrated a potential ergogenic property of S-PS.

### Phosphatidylserine and Mental Stress

Mental and physical stress is closely linked and results in similar physiological responses in the human body. Forty-eight male undergraduate students received either 300 mg S-PS or placebo for 30 days. Mental stress was induced by performing a demanding mathematical task. Phosphatidylserine supplementation resulted in improvements in feeling clear-headed, composed and confident, feeling energetic and elated in a specific sub-section of the young healthy students [10].

Hellhammer et al. investigated the effects of a three week soy lecithin phosphatidic acid and phosphatidylserine complex (PAS) supplementation to a mental and emotional stressor [41]. 400 mg PAS resulted in a pronounced blunting of serum ACTH and cortisol levels as well as positive effects on emotional responses compared to placebo, however, higher doses (600 mg and 800 mg) did not result in the same effects.

Phosphatidylserine has a long history in improving brain functions that tend to decline with age. Numerous clinical trials with PS have showed improvements in memory tasks such as name-face delayed recall, facial recognition, telephone-number recall and misplaced-objects recall [42]. Phosphatidylserine supplementation has been reported to improve long-term memory, long-term recognition, as well as free speech and logic speaking [8]. It has been observed that the interest in the environment and the attention span were increased while loss of motivation, socialization and initiative were reduced [42].

## Conclusion

Plasma choline levels can decrease during intense physical activity of long duration. Where circulating choline concentrations have been demonstrated to decrease during physical activity, choline supplementation has shown benefit in improving performance.

The effective dosage in sport studies is 0.2 g PC (90%) per kg body mass, which equals 2.1 g of choline for an 80 kg athlete. There is no requirement for a loading or maintenance phase and supplementation up to one hour before exercise has been shown to be effective.

Phosphatidylserine has been demonstrated to be an effective supplement for combating exercise-induced stress and preventing the physiological deterioration that accompanies too much exercise (i.e. overtraining). Studies examining athletes involved in cycling, weight training and endurance running suggest that PS might speed up recovery, prevent muscle soreness, improve well-being, and might possess ergogenic properties. Under some circumstances, PS has been reported to suppress cortisol. This might protect muscle membranes against phospholipase damage, which is formed in response to muscle trauma. In addition, PS could increase the rate of glucose transported into muscle cells and, therefore, enhance recovery of muscle glycogen following strenuous exercise.

The effective dosages in sport studies range from 300 to 800 mg PS per day for short-term application (10 -15 days). The effective dosage for mental stress, which is closely related to physical stress, is 300 mg PS per day for 30 days. However, long-term studies on cognitive functions have found significant physiological effects using daily dosages as low as 100 mg PS per day. The lowest effective dose for athletes is unknown.

Many of the richest dietary sources of PC and PS are also high in cholesterol and fat. Egg yolk, meat and internal organs are good sources of phospholipids. Whereas grains, fruits and vegetables are relatively low in PC and PS. Modern Diets are depleted of phospholipids (primarily refined oils and fats, dehulled and cleaned raw materials). These changes in diet (low fat, low Cholesterol), as well as the BSE crises (mad cow disease), have led to a decrease in daily PS and PC consumption. Today's foods contain approximately one third of the phospholipid levels compared to the beginning of last century [7], which could increase the athletes need for proper nutrition and/or supplementation.

## Competing interests

The author(s) declare that they have no competing interests.

## Authors' contributions

All authors contributed equally to this work. All authors have read and approved the final manuscript.
